# Fermented *Gastrodia elata* Bl. Alleviates Cognitive Deficits by Regulating Neurotransmitters and Gut Microbiota in D-Gal/AlCl_3_-Induced Alzheimer’s Disease-like Mice

**DOI:** 10.3390/foods13132154

**Published:** 2024-07-08

**Authors:** Yu Wang, Min Zhao, Chunzhi Xie, Lilang Li, Ling Lin, Qiji Li, Liangqun Li, Faju Chen, Xiaosheng Yang, Juan Yang, Ming Gao

**Affiliations:** 1 State Key Laboratory for Functions and Applications of Medicinal Plants, Guizhou Medical University, Guiyang 550014, China; wang_yu@gmc.edu.cn (Y.W.); luckluckzm@163.com (M.Z.); lililang@gmc.edu.cn (L.L.); 17685222422@163.com (L.L.); leeqiji@126.com (Q.L.); liliangqun2010@163.com (L.L.); chenfaju202212@126.com (F.C.); gzcnp@sina.cn (X.Y.); yangxz2002@126.com (J.Y.); 2 Natural Products Research Center of Guizhou Province, Guiyang 550014, China; 3 College of Food and Biotechnology Engineering, Xuzhou University of Technology, Xuzhou 221018, China; xcz0611@xzit.edu.cn

**Keywords:** *Gastrodia elata* Bl., Alzheimer’s disease, fermentation, neurotransmitter, gut microbiota

## Abstract

Alzheimer’s disease (AD) is a common neurological disease with recognition ability loss symptoms and a major contributor to dementia cases worldwide. *Gastrodia elata* Bl. (GE), a food of medicine–food homology, has been reported to have a mitigating effect on memory and learning ability decline. However, the effect of GE fermented by *Lactobacillus plantarum*, *Acetobacter pasteurianus*, and *Saccharomyces* (FGE) on alleviating cognitive deficits in AD was not studied. Mice were randomly divided into six groups, control, model, donepezil, low, medium, and high doses of FGE, and D-Galactose/Aluminum chloride (D-Gal/AlCl_3_) was used to establish an AD-like mouse model. The results indicated that FGE could improve the production of neurotransmitters and relieve oxidative stress damage in AD-like mice, which was evidenced by the declined levels of amyloid-β (Aβ), Tau, P-Tau, acetylcholinesterase (AchE), and malondialdehyde (MDA), and increased acetylcholine (Ach), choline acetyltransferase (ChAT), and superoxide dismutase (SOD) levels in brain tissue. Notably, FGE could enhance the richness of the gut microbiota, especially for beneficial bacteria such as *Lachnospira* and *Lactobacillus*. Non-target metabolomics results indicated that FGE could affect neurotransmitter levels by regulating amino acid metabolic pathways to improve AD symptoms. The FGE possessed an ameliorative effect on AD by regulating neurotransmitters, oxidative stress levels, and gut microbiota and could be considered a good candidate for ameliorating AD.

## 1. Introduction

Alzheimer’s disease (AD), a type of neurodegenerative disease with insidious onset, is characterized by memory impairment, aphasia, apraxia, agnosia, and executive and spatial recognition dysfunction [1,2,3]. There are over 55 million patients with dementia worldwide, according to the “*World Alzheimer’s Disease 2023 Report*” released by the Alzheimer’s Association International (ADI). Even more concerning, the number of patients with dementia is expected to reach 139 million by 2050, with the greatest increases in low- and middle-income countries, of which about 60–70% will be patients with AD [4]. It seriously affects the quality of patient life and is a threat to human health. Unfortunately, despite several decades of research, the pathogenesis of AD is still not fully clear. Recent studies revealed that AD is caused by various pathogenesis factors and involves many pathological mechanisms [5]. The imbalanced production and clearance of amyloid-β (Aβ) is the initial factor of neuronal degeneration and dementia. The excessive Aβ oligomers between brain neurons may accelerate neurodegeneration and then induce Tau hyperphosphorylation, which is one of the main pathogens for the occurrence of AD [6,7,8]. Tau protein, a microtubule-related protein, is mainly distributed in the axons of neurons in the central nervous system. Excessive phosphorylation of Tau protein causes the loss of the ability to bind to microtubules, resulting in microtubule deaggregation and axon transport obstruction, forming Tau aggregates, which then cause the apoptosis of nerve cells and lead to the occurrence of AD [9].

Current studies have confirmed that the gut microbiota, immune system, intestinal mucosal barrier, blood–brain barrier, and the “microbial–gut–brain” axis of the human body all change during aging, which further influences the composition of intestinal flora, metabolites, and the associated inflammatory state of the body [10,11]. The activated neuroimmune–inflammatory response further promotes Aβ production, which then induces and aggravates AD. The composition of intestinal flora in AD patients was obviously different from that of non-AD elderly people. At the phylum level, the abundance of *Bacteroidetes* increased while that of *actinomyces* decreased. At the genus level, the number of *Bacteroides* and *Ruminococcus* increased while that of *Clostridium trichoides* decreased. These results indicated that an increase in pro-inflammatory bacteria in AD patients, and the change in cognitive function may be closely linked to the peripheral systemic inflammation caused by intestinal pro-inflammatory bacteria [12]. Xu et al. found that yeast β-glucans, a type of polysaccharide, prominently shape the intestinal flora, produce SCFAs, and could be a novel dietary supplement to prevent early-stage pathologies associated with AD [13]. Reshaping the balance of intestinal flora and reducing the production of abnormal metabolites of intestinal flora, Aβ deposition, and Tau hyperphosphorylation could be a potential effective strategy to relieve AD symptoms.

*Gastrodia elata* Bl. (GE), an edible and medicinal plant, is used as a traditional functional food to improve sleep and relieve headaches, with a long history according to the *Compendium of Materia Medica*. People living in the Guizhou and Yunnan provinces of China treated GE as a food to cook soup. In 2019, the Chinese government officially announced that GE can be used as both medicine and food [14,15]. Previous studies have shown that the main components of GE, phenols and glycosides, could effectively improve the central nervous system damage and ameliorate the decline of memory and learning ability caused by AD, depression, and Parkinson’s disease [16]. Notably, GE could positively modulate the gut microbiota and pathological changes in AD-related proteins to protect against cognitive impairment [17,18]. 

Microbial fermentation has been used to process food and traditional Chinese medicine for centuries in China with high efficiency, low toxicity, and safety features, and has played a vital role in the production and development of human society [19]. However, studies about the mitigative effect of fermented GE on AD have not been performed. Our previous study confirmed that FGE was associated with better improvement in an animal behavior test than the GE group in AD-like mice (Figure S1). Thus, this study aimed to clarify the anti-AD effects of FGE via regulating neurotransmitters and the gut microbiota in D-Gal/AlCl_3_-induced AD-like mice.

## 2. Materials and Methods

### 2.1. Preparation of FGE and Experimental Animals 

#### 2.1.1. Preparation of FGE

GE powder was mixed with sterile water at a ratio of 1:20 (*m*/*v*), and then, 8% white sugar, 0.02% cellulose, starter (0.3%, *Lactobacillus plantarum* PL15A (Thankcome Bio, Suzhou, China), *Acetobacter pasteurianus* AS141 (Yishui Jinrun Bio, Yishui, China), and *Saccharomyces cerevisiae* BV818 (Angel, Shenzhen, China) at a ratio of 1:1:1 (*w*/*w*/*w*)) were added. The mixture was cultured at 37 °C in a fermenter for 30 days, and the liquid supernatant was boiled and centrifuged at 4000 rpm for 5 min to remove the residue.

#### 2.1.2. Animal Experiments

SPF-grade KM mice (male, 25 ± 2 g, 5–6 weeks) were provided by Liaoning Changsheng Biotechnology Co., Ltd. (Changchun, China, SCXK-2015-0001). The mice were housed in a standard animal feeding center at 23–25 °C with 55–60% humidity and were fed adaptively for one week. All experimental procedures were approved by the Animal Care and Use Committee of the Guizhou Medical University (No. 2304037). The mice were randomly divided into six groups (*n* = 10): control, model, donepezil (1 mg/kg·bw), FGE-L (120 mg/kg·bw), FGE-M (240 mg/kg·bw), and FGE-H (720 mg/kg·bw) groups. The intragastric administration of D-galactose (D-Gal, 120 mg/kg·bw) and aluminum trichloride (AlCl_3_, 40 mg/kg·bw) was conducted in the morning each day for seven weeks to establish an AD-like animal model. The donepezil and FGE groups were administered with the related samples (0.2 mL/d) in the afternoon. The control and model groups received an equal volume of normal saline. Intragastric drug administration was adopted, and the experiments were conducted for 42 days.

### 2.2. Behavioral Tests

#### 2.2.1. Shuttle Box Test

The mice in each group were allowed to move freely in the shuttle box and given 2 min to adapt to the environment. Then, the mice were given a certain degree of light, electrical, and sound stimulation, with each cycle interval being 15 s, and trained for 30 cycles daily for five days. In the conditioned stimulus period, the mice escaped to the other side through the middle channel, which was recorded as an active escape. In the unconditioned stimulus period, the mice shuttled to the opposite atrial ventricle, which was a passive escape, and the numbers of active escapes were recorded. During the memory test, each mouse was placed in the left ventricle to face the wall and received 10 s of beeping sound and 10 s of foot stimulation, with a 15 s interval for each cycle, for a total of 30 cycles. After each mouse completed the test, 75% alcohol was used to clean the surfaces of the instrument to remove the odor of the previous mouse.

#### 2.2.2. Open Field Test

The motion of mice was recorded with a camera for 5 min in an open-field device box, and the total movement distance of the mice was recorded. Similarly, the residual odor of mice in the box was cleaned with 75% alcohol to prevent interference by residual odors.

### 2.3. Determination of Neurotransmitters and Oxidative Stress Indexes

The hippocampus area of brain tissue was mixed with PBS buffer solution (*w*/*v* = 1:9), and then the mixture was homogenated by a tissue homogenizer and centrifuged for 15 min at 10,000 rpm at 4 °C to obtain the supernatant. The contents of Aβ, Tau, P-Tau, acetylcholine (Ach), acetylcholinesterase (AchE), acetylcholintransferase (ChAT), and malondialdehyde (MDA) and the activity of superoxide dismutase (SOD) in brain tissue were investigated using correlated assay kits according to the manufacturer’s instructions.

### 2.4. H&E Staining 

The fixed brain tissue was infiltrated with paraffin and sliced with a slicer, and then the paraffin sections were dewaxed with xylene solution. The following procedures were conducted for staining and dehydration: cleaning solution I for 10 min, cleaning solution II for 10 min, cleaning solution III for 10 min; gradient alcohol dewaxing hydration: dehydration with 100% ethanol for 15 min, dehydration with 95% ethanol for 5 min, dehydration with 80% ethanol for 5 min, rinsing with double steaming water twice, 5 min/times, staining with hematoxylin for 6 min, rinsing with pure water, differentiating liquid for 30 s, returning to blue water for 10 min, staining with eosin for 2 min, and rinsing with pure water for 10 min. And then, ethanol dehydration, xylene transparency, and the sealed sheet were assessed.

### 2.5. Immunohistochemistry Analysis

The brain tissue was fixed in 4% paraformaldehyde for 3 h and dehydrated with alcohol and xylene. Xylene I, II, and III solutions were used for dewaxing for 10 min. Antigen repair solution (200 mL) was heated in a microwave oven, paraffin sections were added for 5 min, and the sections were washed with PBS thrice. The sample was allowed to inactivate after adding peroxidase and a serum sealer for 10 min at room temperature; the primary antibody was added and incubated overnight at 4 °C. Secondary antibodies were added for 10 min at room temperature after the samples were rewarmed. Antibiotin–peroxidase solution was added and incubated at room temperature for 10 min. Then, the DAB reaction was performed at room temperature for 5 min, and hematoxylin was added for 3–4 min, followed by PBS and washing with 95% ethanol for 10 min. The samples were immersed in xylene I, II, and III solutions for 5 min each and sealed.

### 2.6. Gut Microbiota DNA Extraction and Analysis of Gut Microbiota

Cecal contents were collected from mice for DNA extraction using an E.Z.N.A.^®^ Soil DNA Kit (Omega Bio-tek, Norcross, GA, USA) according to the manufacturer’s protocols. TBS-380 and NanoDrop2000 were applied to determine the concentration and purity of the extracted DNA from the intestinal canal, respectively. Then, Covaris M220 (Gene Company Limited, Hong Kong, China) was used to clip the DNA extract to an average size of approximately 400 bp for paired-end library construction. Paired-end sequencing was conducted on an Illumina Novaseq 6000 (Illumina Inc., San Diego, CA, USA) at Majorbio Bio-Pharm Technology Co., Ltd. (Shanghai, China). 

### 2.7. UPLC-MS Analysis 

The FGE was dissolved in HPLC-grade methanol and filtered through a 0.22 μm microporous membrane to perform LC-MS analysis. The LC-MS analysis was conducted in a UPLC-Q-Exactive MS (Thermo Scientific, Waltham, MA, USA) with a Waters Aaquity UPLC HSS T3 column (100 mm × 2.1 mm, 1.8 μm) by using gradient elution at a flow rate of 0.3 mL/min (5% B (0 min)—5% B (2 min)—95% B (42 min)—95% B (47 min)—5% B (47.1 min)—5% B (50 min); A: H_2_O + 0.1% formic acid; B: acetonitrile + 0.1% formic acid). The temperature of the column oven was set at 60 °C and the injection volume at 10 μL. The fractions were analyzed in data-dependent acquisition (DDA) mode: The full MS survey scan was performed for 100 ms in the range of 100–1500 Da, and the gradient of collision energy was set as 35 eV. Compounds were identified by comparison with the MS spectral data from previously isolated compounds and the mzValut (Thermo Fisher Scientific, Waltham, MA, USA) database.

### 2.8. Statistical Analysis

Robust regression analysis was conducted using GraphPad Prism 8 (GraphPad Prism, La Jolla, CA, USA). One-way analysis of variance was used to analyze the differences between groups. All data were presented as mean ± SD. Tukey’s post hoc tests were used to analyze the differences between groups. Statistical significance was set at *p* < 0.05. 

## 3. Results

### 3.1. Identification of Main Components in FGE

**Figure 1:**
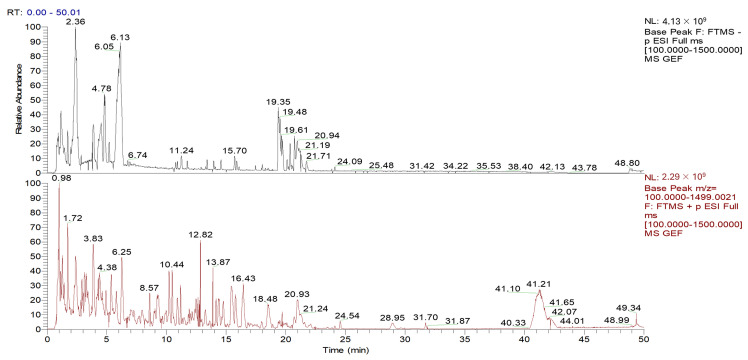
UPLC/Q-TOF-MS/MS chromatogram of the FGE in positive ionization mode.

UPLC-HR/MS was applied to identify the main components of FGE, and 67 compounds were identified as shown in Figure 1 and Table S1. Among them, 42 compounds were identified as phenolic compounds, including gastrodin, p-hydroxybenzyl alcohol, p-hydroxybenzoic acid, p-hydroxybenzaldehyde, parishin D, etc. Meanwhile, 16 organic acids were confirmed, including fumaric acid, citric acid, glucuronic acid, gluconic acid, etc.

### 3.2. The Effect of FGE on Cognitive Function in AD-like Mice

**Figure 2:**
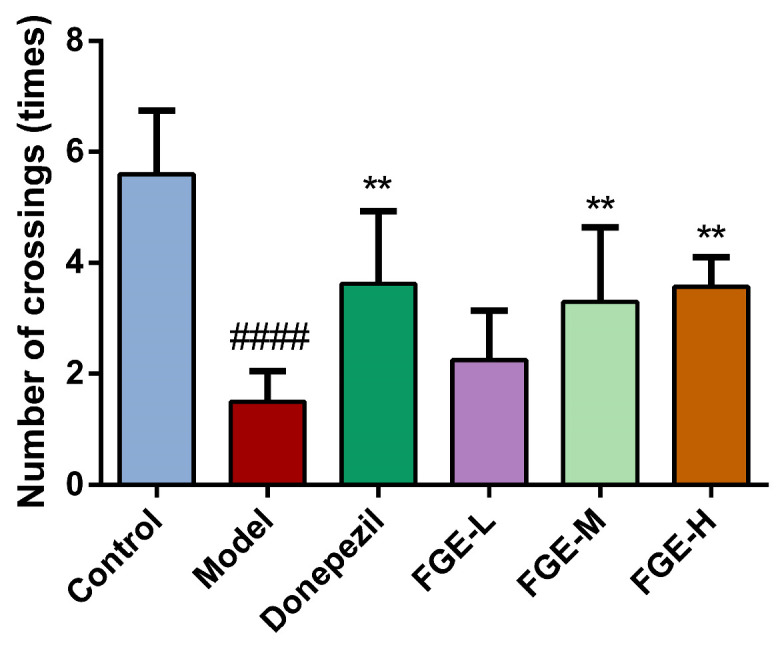
Effects of FGE on number of crossings of AD-like mice. FGE-L (120 mg/kg b. w.), FGE-M (240 mg/kg b. w.), and FGE-H (720 mg/kg b. w.). Data are expressed as means ± SD (*n* = 10) of six independent experiments. ^####^ *p* < 0.0001 compared with control group, ** *p* < 0.05 compared with model group.

The shuttle box test is generally used to train the active conditioned reflex of the mouse brain, which could represent changes in the learning and memory ability of animals. As illustrated in Figure 2, the number of active escapes in the model group was significantly reduced compared with the control group (*p* < 0.01), indicating that an AD-like animal model was successfully established. After FGE administration, the number of active escapes was significantly higher than that in the model group in a dose-dependent manner (*p* < 0.01). These results indicated that the FGE groups could effectively improve the learning ability, memory loss, and inactive behavior of AD-like mice. 

### 3.3. The Effect of FGE on Anxiety-like Behavior in AD-like Mice

**Figure 3:**
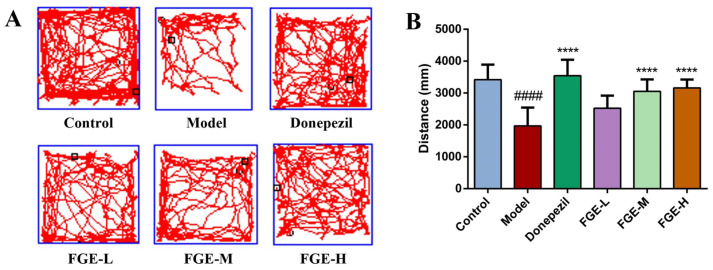
(**A**,**B**) Effects of FGE on total movement distance of AD-like mice. FGE-L (120 mg/kg b. w.), FGE-M (240 mg/kg b. w.), and FGE-H (720 mg/kg b. w.). Data are expressed as means ± SD (*n* = 10) of six independent experiments. ^####^ *p* < 0.0001 compared with control group, **** *p* < 0.0001 compared with model group.

The total distance of movement in an open-field experiment is an important factor for evaluating the memory ability and behavior activity of animals [20]. As demonstrated in Figure 3, the total movement distance was significantly reduced in the model group compared with that in the control group (*p* < 0.01), indicating that an AD-like animal model was successfully established. Notably, the total movement distance of mice in the donepezil and FGE groups was significantly increased compared with the model group (*p* < 0.01). Accordingly, administrating FGE could improve the behavioral activity and cognition of AD-like mice.

### 3.4. The Effect of FGE on the Levels of Neurotransmitters in Brain Tissue of AD-like Mice

**Figure 4:**
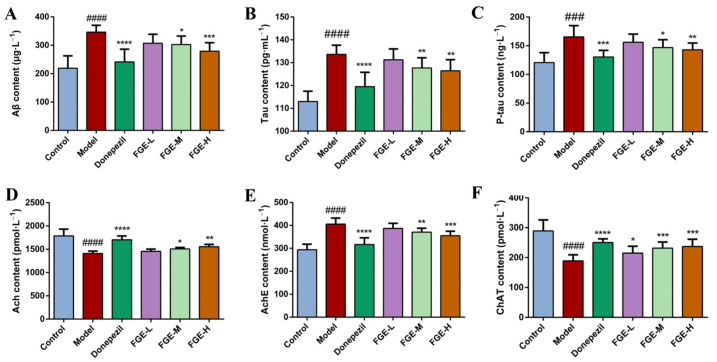
Effects of FGE on the production of neurotransmitters in the brain tissue of AD-like mice. (**A**) Aβ; (**B**) Tau; (**C**) P-Tau; (**D**) Ach; (**E**) AchE; (**F**) ChAT; FGE-L (120 mg/kg b. w.), FGE-M (240 mg/kg b. w.), and FGE-H (720 mg/kg b. w.). Data are expressed as means ± SD (*n* = 10) of six independent experiments. ^###^ *p* < 0.001, ^####^ *p* < 0.0001 compared with control group; * *p* < 0.01, ** *p* < 0.05, *** *p* < 0.001, **** *p* < 0.0001 compared with model group.

The damage of neuronal cells in brain tissue is generally caused by an abnormal increase in Aβ, which leads to the misfolding and assembly of Tau proteins throughout the cortex, eventually leading to nervous system failure, neurodegeneration, and cognitive decline. As displayed in Figure 4, the contents of Aβ, Tau, P-Tau, and AchE were significantly increased, while Ach and ChAT sharply decreased in the model group compared to the control group (*p* < 0.05). Interestingly, treatment with FGE could significantly improve the secretion of Aβ, Tau, P-Tau, and AchE and increase Ach and ChAT contents in the brain tissue. FGE could positively regulate neurotransmitter contents in AD-like mice.

### 3.5. The Effect of FGE on the Content of Antioxidants in AD-like Mice

**Figure 5:**
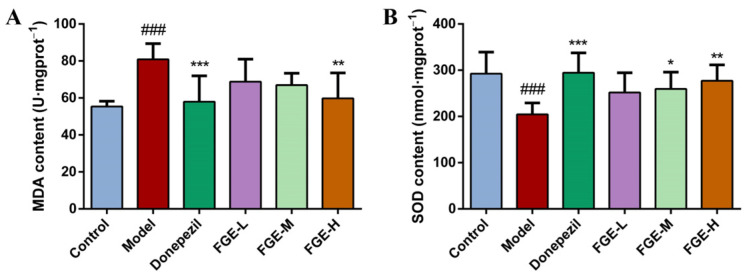
Effects of FGE on the levels of antioxidants in the brain tissue of AD-like mice. (**A**) MDA; (**B**) SOD; FGE-L (120 mg/kg b. w.), FGE-M (240 mg/kg b. w.), and FGE-H (720 mg/kg b. w.). Data are expressed as means ± SD (*n* = 10) of six independent experiments. ^###^ *p* < 0.001 compared with control group; * *p* < 0.05, ** *p* < 0.05, *** *p* < 0.001 compared with model group.

Oxidative stress (OS) is a series of adaptive reactions caused by an imbalance between reactive oxygen species and the antioxidant system of the body [21]. It interferes with the normal redox state of cells and damages proteins, lipids, and nucleic acids. The imbalance of OS drives AD, PD, atherosclerosis, heart failure, and cancer. As depicted in Figure 5, the MDA content was significantly increased (*p* < 0.01) and the SOD content was decreased in the model group compared to the control group. Compared with the model group, the levels of MDA and SOD were decreased and increased in the brain tissue in a dose-dependent manner in the donepezil and FGE groups.

### 3.6. The Effect of FGE on the Damage of Neuronal Cells in the Hippocampus of AD-like Mice

**Figure 6:**
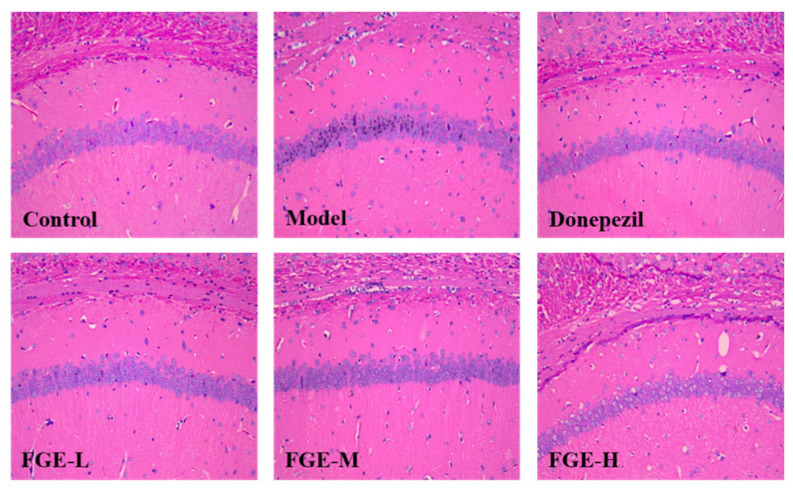
Effects of FGE on hippocampal neurons in CA1 of AD-like mice. Sections were stained with hematoxylin and eosin and observed using Leica microscope (*n* = 3). FGE-L (120 mg/kg b. w.), FGE-M (240 mg/kg b. w.), and FGE-H (720 mg/kg b. w.).

As displayed in Figure 6, the number of nerve cells was reduced, the arrangement was irregular, the integrity of the pyramidal cell layer was lost, and the cell body and nucleus were shrunken in the model group compared with the control group. Treatment with FGE improved neuronal cell damage, decreased cell shrinkage, improved cell morphology, increased cell layers and neat cell arrangement, and significantly increased cytoplasm and nucleus clarity compared with the model group. Accordingly, FGE could improve the symptoms of AD-like mice by protecting nerve cells in the brain tissue.

### 3.7. The Effect of FGE on the Protein Expression of Aβ in the Hippocampus of AD-like Mice

**Figure 7:**
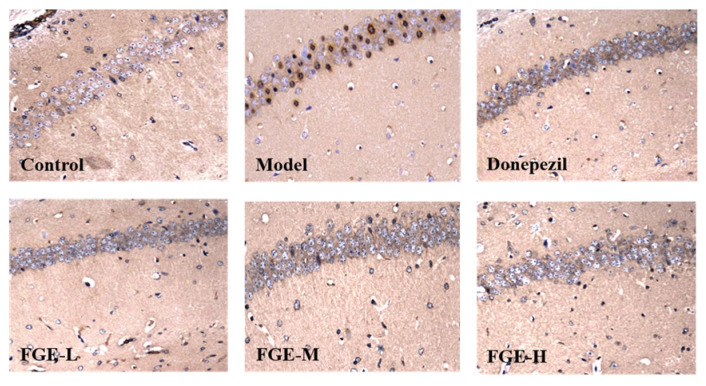
Effects of FGE on the content of Aβ in the brain tissue of AD-like mice (*n* = 3). FGE-L (120 mg/kg b. w.), FGE-M (240 mg/kg b. w.), FGE-H (720 mg/kg b. w.).

Aβ, a type of amyloid protein in the body, has an important effect on cognition, learning, and memory, which is the main contributing factor to AD pathogenesis. Aβ deposition was significantly increased in the model group compared with the control group, indicating that the production and degradation system of Aβ in the brain was disturbed, causing the brain Aβ not to be degraded. Remarkably, Aβ accumulation in the hippocampus of the brain tissue was significantly reduced in FGE-treated groups compared with the model group, indicating that FGE could improve AD symptoms by degrading the content of Aβ in brain tissue (Figure 7).

### 3.8. The Effect of FGE on the Abundance of Intestinal Flora in AD-like Mice

**Figure 8:**
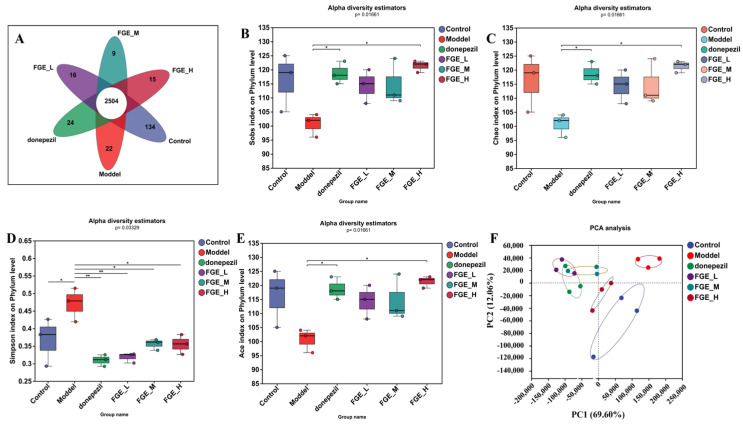
Effects of FGE on the gut microbiota in AD-like mice. Venn diagram of the OTUs (**A**); beta diversity indicated by Sobs (**B**), Chao (**C**), Simpson (**D**), Ace (**E**), and PCoA (**F**) analysis (*n* = 3); * *p* < 0.05, ** *p* < 0.05, compared with model group.

To investigate the effect of FGE on the gut microbiota composition, the 16S rRNA gene sequences of 18 samples from six groups were sequenced using Majorbio HiSeq (Shanghai Majorbio Biomedical Technology Co., Ltd., Shanghai, China). The taxonomic composition at the genus level was characterized by operational taxonomic units (OTUs). At the genus level, the Venn diagram indicated that there were 2504 shared OTUs among the six groups and 134, 22, 24, 16, 9, and 15 unique OTUs in the control, model, donepezil, FGE-L, FGE-M, and FGE-H groups, respectively (Figure 8A). Alpha diversity estimations and PCA analyses indicated that the intestinal microbiota was separated in these six groups (Figure 8B–F). After treatment with FGE, the gut microbiota was similar to that in the control group. 

**Figure 9:**
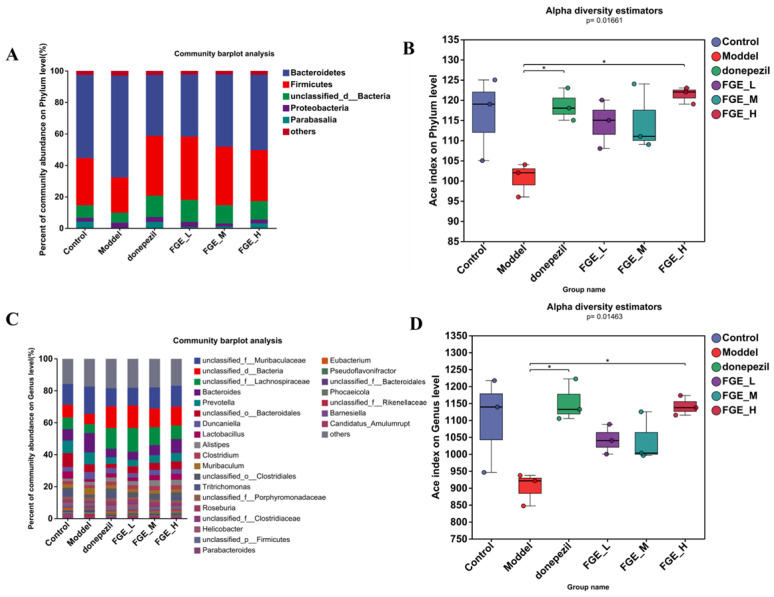
Relative abundance of bacteria at the phylum and genus levels. Histogram of relative abundance at the phylum level (**A**); Alpha diversity determined by Ace diversity index at the phylum level (**B**); Histogram of relative abundance at the genus level (**C**); Alpha diversity determined by Ace diversity index at the genus level (**D**). One-way ANOVA with LSD *t*-test was applied to evaluate significant differences between the groups; * *p* < 0.05 (*n* = 3).

**Figure 10:**
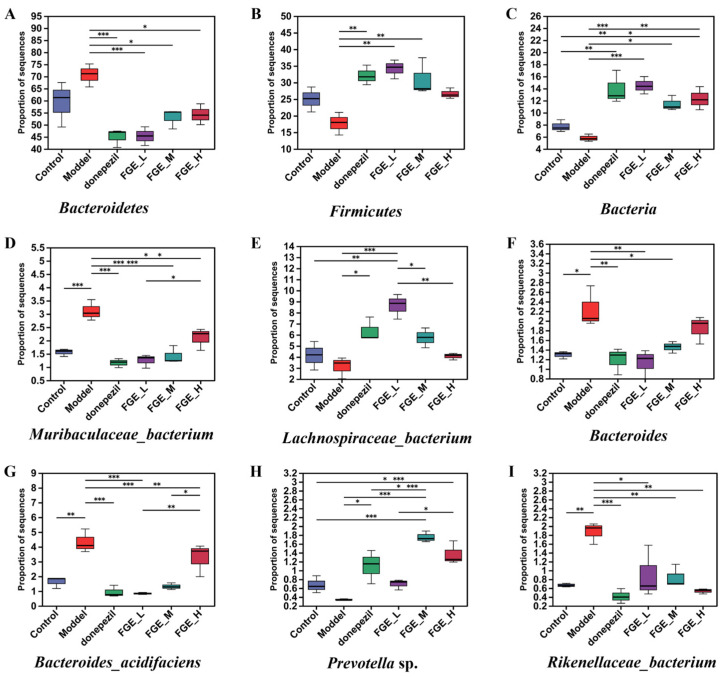
Abundance of bacterial taxa at different taxonomic levels (*n* = 3). (**A**) Phylum Bacteroidetes; (**B**) phylum Firmicutes; (**C**) phylum Bacteria; (**D**) species *Muribaculaceae*_*bacterium*; (**E**) species *Lachnospiraceae*_*bacterium*; (**F**) species *Bacteroides*; (**G**) species *Bacteroides*_*acidifaciens*; (**H**) species *Prevotella* sp.; (**I**) species *Rikenellaceae*_*bacterium*. * *p* < 0.05, ** *p* < 0.05, *** *p* < 0.001 compared with model group.

At the phylum level, Bacteroidetes, Firmicutes, Bacteria, Proteobacteria, and Parabasalia were the most abundant, whereas the abundance of Firmicutes and Bacteroidetes was markedly decreased and increased in the model group compared with the control group (Figure 9A,B). Intriguingly, FGE treatment could increase the abundance of Firmicutes, and decrease the abundance of Bacteroidetes. The imbalance of the two phyla can affect glucose metabolism in the brain tissue, and abnormal glucose metabolism can lead to increased Aβ deposition, which ultimately increases the risk of AD. At the species level, the abundance of *Muribaculaceae*, *Rikenellaceae*, and *Bacteroides* was obviously increased, and the abundance of *Lachnospiraceae* and *Prevotella* sp. was decreased in the model group compared with the control group (Figure 10). Prominently, treatment with FGE could improve the abundance of *Lachnospiraceae* and decrease the abundance of *Muribaculaceae* and Bacteroidaceae compared with the model group. The present study confirmed that the abundance of *Lachnospiraceae* was decreased in the intestinal canal of patients with AD, indicating that treatment with FGE could ameliorate the symptoms of AD. 

### 3.9. FGE Regulates the Imbalance of Amino Acid Metabolism

**Figure 11:**
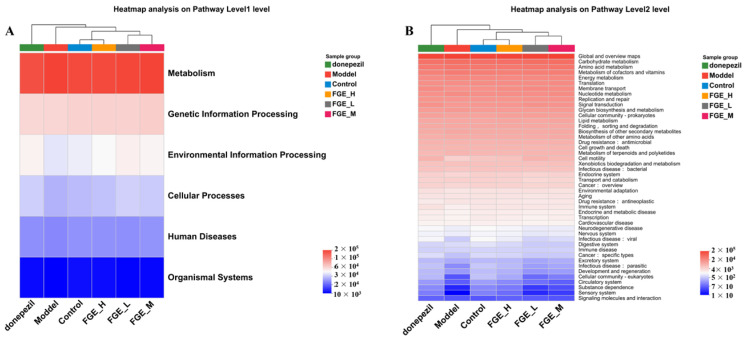
Heat map describing the primary and secondary metabolic function of intestinal microorganisms in AD-like mice (*n* = 3).

The intestinal flora is regulated by host genes, immune response, and dietary factors, and its metabolic and immune potential determines its importance in host health and disease [22]. Therefore, targeting the gut microbiota and its associated metabolic pathways is an effective strategy for treating diseases. In this study, the metabolic pathways in the six groups were analyzed using the KEGG database. As demonstrated in Figure 11A, metabolism, genetic information processing, and environmental information processing contributed to the top three level-1 pathways in these six groups. Further analysis of the KEGG secondary metabolic map indicated that the global and overview maps, carbohydrate metabolism, and amino acid metabolism are the top 3 metabolic functions among the 38 metabolic functions. As demonstrated in Figure 11B, the greatest difference in a metabolic pathway is amino acid metabolism among the six groups. AD is a multi-mechanism metabolic disorder closely linked to energy metabolism, in which amino acid metabolism plays an important role. Moreover, studies have found that amino acid levels have been identified as potential biomarkers for patients with AD. Consequently, we speculate that FGE may improve AD by intervening in the amino acid metabolism.

### 3.10. The Effect of FGE Treatment on Neurotransmitters by Regulating Gut Microbiota

**Figure 12:**
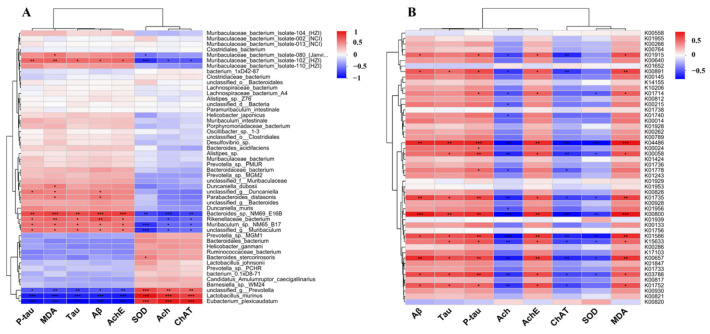
Heat map describing the correlation of the abundances of key bacterial genera at species levels and AD-related parameters (**A**); the correlation of the amino acid metabolic pathways and AD-related parameters (**B**). * *p* < 0.05, ** *p* < 0.01, *** *p* < 0.001.

Amino acid metabolism, the most varied metabolic pathway, is linked to neurotransmitter and oxidative stress levels. As illustrated in Figure 12A, the intestinal flora of mice in different groups was significantly different at the genus level. The red and blue colors indicate positive and negative correlations with the relevant indicators, respectively. Lactobacillus, *Rikenellaceae*, Parabacteroides, Eubacterium, Parasutterella, and *Rikenellaceae* were significantly correlated with Aβ, Tau, P-Tau, Ach, AchE, ChAT, SOD, and MDA contents. Consequently, the structure of the intestinal flora has an impact on the level of neurotransmitters and oxidative stress, indicating that treatment with FGE could regulate the brain–nervous system by maintaining the intestinal flora structure of the body to improve the effect of AD.

### 3.11. Correlation Analysis between Amino Acid Metabolism and Oxidative Stress Level in AD-like Mice

Amino acid metabolism is the most abundant differential metabolic pathway involved in the synthesis of proteins, including neurotransmitters. Our results indicated that the metabolic pathways of K00891, K04486, K00058, K01735, K00800, K01586, K00657, K03786, and K01752 are crucial for the synthesis of neurotransmitters and OS-related indicators (Figure 12B). Further studies have indicated that the overactive biosynthesis pathway of phenylalanine, tyrosine, and tryptophan could lead to AD development and exacerbation. Phenylalanine could be converted into hippuric acid (HA) by an imbalance of intestinal flora, and excessive HA causes neuroinflammation and affects the level of neurotransmitters and oxidative stress in the body. Remarkably, FGE could improve AD by interfering with the HA synthesis by regulating the amino acid pathway.

## 4. Discussion

Phenolic compounds are the main active ingredients in GE, especially gastrodin, which has been widely used and studied, and has obvious application effects in neurological diseases such as stroke, epilepsy, and AD [23]. In our study, only parishin D was identified from FGE, indicating that the microbial fermentation could degrade the parishin to gastrodin and then increase its clinical efficacy for AD [24]. In addition, microbial fermentation can improve the taste and flavor of products, such as ethyl succinate, a flavoring agent, which was identified from FGE and is mainly produced by *Saccharomyces cerevisiae*. The dynamic changes in flavor components, including organic acids, alcohol, lipid, and ether organic acids, during the fermentation have been studied previously [25]. 

The unbalanced production and clearance of Aβ are the initial factors in neuronal degeneration and dementia. The plaques formed between brain neurons due to abnormal levels of Aβ protein are neurotoxic and lead to neuronal degeneration. Aβ, a large molecule of protein from the fatty membrane of nerve cells, has sticky chemical properties and is a major component of amyloid plaques [26]. Certain types of Aβ, such as Aβ38/40/42, coagulate to form small clumps of protein that accumulate to form plaques. This sick Aβ can infect normal Aβ, thereby increasing plaque formation. Tau protein is a microtubule-related protein, and one of its main functions is to maintain the stability of axonal microtubules and ensure normal brain function. In our study, FGE treatment could significantly inhibit Aβ deposition in the hippocampal region by protecting the mechanism of Aβ movement in neurons.

The defective and hyperphosphorylated Tau protein can bind to microtubules and cause neurofibrillary tangles. In summary, the formation of Aβ plaques and neurofibrillary tangles of Tau proteins accelerates damage to healthy neurons. The deposition of Aβ plaque can trigger a series of chain reactions, which lead to the misfolding and assembly of Tau protein in the cell and then spread the disease to the entire nerve circuit and cortex, eventually leading to nervous system failure and cognitive decline [27].

The cholinergic hypothesis is the first hypothesis that attempts to explain AD pathogenesis. It is believed that AD occurrence is due to defects in neurotransmitters in the brains of patients with AD, resulting in damage to cholinergic neurons. This hypothesis suggests that a decrease in AChE and ChAT activity is the main reason for the decrease in Ach concentration and cholinergic activity [28]. The acetylcholine receptor antagonist scopolamine could cause cognitive dysfunction, increase the production of Aβ, and inhibit the activity of α-secreting enzyme [29]. Interestingly, we found that FGE positively regulated neurotransmitter levels in the brain tissue of AD-like mice, such as Aβ, Tau, P-Tau, Ach, AchE, and ChAT [30].

Studies have found that many diseases, such as cerebral ischemia, epilepsy, schizophrenia, PD, and AD neuronal degeneration, are closely associated with excessive free radicals [31]. An increased degree of OS results from the production of a large number of lipid peroxides, such as MDA, originating from the oxidation of intracellular lipids [32]. SOD is an important antioxidant enzyme that removes superoxide anion free radicals in organisms, which can protect cells from the damage caused by oxygen free radicals. SOD and MDA, as important indexes to evaluate the antioxidant capacity and oxidative capacity of oxidative stress, respectively, are jointly applied in the field of disease research [33]. Amin et al. reported that SOD and MDA could be markers of OS to study the association of benzene exposure and insulin resistance in children [34]. In this study, we found that FGE treatment could reduce the MDA levels and increase the SOD content, indicating that FGE could regulate the imbalance of OS in the body. The “microbial–gut–brain” axis is a two-way communication pathway between the gut and the brain conducting neural, immune, endocrine, and metabolic signals [35,36]. Abnormal composition of the gut microbiota has been found in several neuropsychiatric disorders, such as autism spectrum disorders, depression, PD, and AD. Recent studies have disclosed that intestinal flora plays a key role in regulating intestinal and brain functions [37]. Exploring the relationship between intestinal flora and AD can provide new strategies and ideas for preventing and treating AD. Ferreiro et al. found that disturbances in the gut microbiota occur in the preclinical phase of AD and are closely linked to the formation of pathological markers of Aβ and Tau proteins [38]. *Firmicutes* play an important role in host intestinal homeostasis through short-chain fatty acid synthesis, normalizing intestinal permeability, and participating in brain–gut axis regulation [39]. *Lactobacillus*, a Gram-positive bacterium belonging to Firmicutes, could produce acetate, and lactic acid, gamma-aminobutyric acid and convert tryptophan metabolism into neurotransmitters such as 5-HT [40]. Studies have found that low levels of 5-HT can affect the signaling of other neurotransmitters in brain tissue and increase the accumulation of Aβ plaques in the brain [41]. Interestingly, the proportion of *Lactobacillus* in intestinal flora sharply increased after treatment with FGE compared with the model group. In addition, our study found that the abundance of *Lachnospira* markedly increased in the FGE groups. Studies have found that *Lachnospira* is a potentially beneficial bacterium in the human gut, which is involved in the metabolism of various carbohydrates and is the main source of energy for the host [42]. Moreover, the increased abundance of *Lachnospira* could promote the growth of *Bacteroides* in the intestinal canal. Chen et al. found that the levels of *Bacteroides* and *Lachnospira* were reduced, and those of *Prevotella* were increased in patients with AD compared with healthy controls, indicating that FGE could restore intestinal homeostasis [43].

Changes in the composition of gut microbes are associated with host physiology and human diseases, such as diabetes, heart disease, and depression, through excessive production of short-chain fatty acids such as butyric acid, propionic acid, succinic acid, and acetic acid which affect the host metabolism [44,45]. Cui et al. reported that the glutamine/glutamate and glycine/serine/threonine metabolism pathways were abnormally altered in the serum of patients with AD [46].

The phenylalanine, tyrosine, and tryptophan metabolic pathways have a positive contribution in regulating neurotransmitters in serum, such as Aβ, Tau, P-Tau, Ach, AchE, ChAT, SOD, and MDA, which were confirmed in this study. Moreover, abnormal levels of phenylalanine could be converted into hippuric acid (HA) by intestinal microorganisms, and excessive HA causes neuroinflammation and affects the level of neurotransmitters and oxidative stress in the body. Tryptophan metabolism is regulated by intestinal microorganisms, and its metabolites possess immune, metabolic, and neuroregulatory functions, which have become therapeutic targets for various diseases. Trp metabolism has been implicated in several neurodegenerative diseases, including Huntington’s disease (HD), AD, amyotrophic lateral sclerosis (ALS), and PD. Trp metabolism, a potential mechanism of neurodegeneration, includes excitatory toxicity through TRP-dependent protein toxicity, accumulation of Trp metabolites, and energy imbalance due to NAD+ depletion [47].

## 5. Conclusions

In this study, we found that FGE improved behavioral cognitive deficits in AD-like mice treated with GE powder and could ameliorate the damage to neuronal cells in the hippocampus by positively regulating neurotransmitters such as Aβ, Tau, P-Tau, AchE, Ach, and ChAT. Moreover, analysis of the gut microbiota composition found that FGE could enhance the abundance of probiotics, such as *Lachnospira* and *Lactobacillus*, which were correlated with the production of neurotransmitters. Furthermore, we confirmed that the amino acid metabolism pathway is the most differential metabolic pathway that is closely correlated with AD development. As a result, microbial-based fermentation can be a potential strategy for treating GE to achieve greater medicinal value. In addition, FGE could be a new potential source for treating AD by regulating the gut microbiota. 

## Data Availability

The original contributions presented in the study are included in the article/Supplementary Materials, further inquiries can be directed to the corresponding author.

## Supplementary Materials

The following supporting information can be downloaded at: https://www.mdpi.com/article/10.3390/foods13132154/s1, Figure S1: Effects of GE powder and FGE on number of crossings of AD-like mice; Figure S2: Effects of GE powder and FGE on total movement distance of AD-like mice; Figure S3: LDA Effect Size analysis of community differences between groups; Table S1: Chemical composition identification of FGE using HR-LC/MS in positive mode.
