# Supplementary material for: Fermented Gastrodia elata Bl. Alleviates Cognitive Deficits by Regulating Neurotransmitters and Gut Microbiota in D-Gal/AlCl3-Induced Alzheimer’s Disease-like Mice
Source: Foods. 2024 Jul 8;13(13):2154. doi: 10.3390/foods13132154 (PMC11241452; doi:10.3390/foods13132154)
Supplement: Supplementary file 1 [file foods-13-02154-s001.zip › foods-3035652-supplementary.pdf]

Fermented *Gastrodia elata* Bl. Alleviates Cognitive Deficits by Regulating Neurotransmitters and Gut Microbiota in D-Gal/$AlCl_3$-Induced Alzheimer's Disease-Like Mice

Yu Wang [1, 2], Min Zhao [1,2], Chunzhi Xie [3], Lilang Li [1,2], Ling Lin [1,2], Qiji Li [1,2], Liangqun Li [1,2], Faju Chen [1,2], Xiaosheng Yang [1,2], Juan Yang [1,2] and Ming Gao [1,2,*]

[1] State Key Laboratory for Functions and Applications of Medicinal Plants, Guizhou Medical University, Guiyang, 550014, China;

[2] Natural Products Research Center of Guizhou Province, Guiyang, 550014, China

[3] College of Food and Biotechnology Engineering, Xuzhou University of Technology, Xuzhou, 221018, China

* Corresponding author: gaoming@gmc.edu.cn ;

Tel: +86 18345701431

Contents

Figure S1: Effects of GE powder and FGE on number of crossings of AD-like mice..3
Figure S2: Effects of GE powder and FGE on total movement distance of AD-like mice..3
Figure S3: LDA Effect Size analysis of community differences between groups..4
Table S1: Chemical composition identification of FGE using HR-LC/MS in positive mode…………………………………………………..5

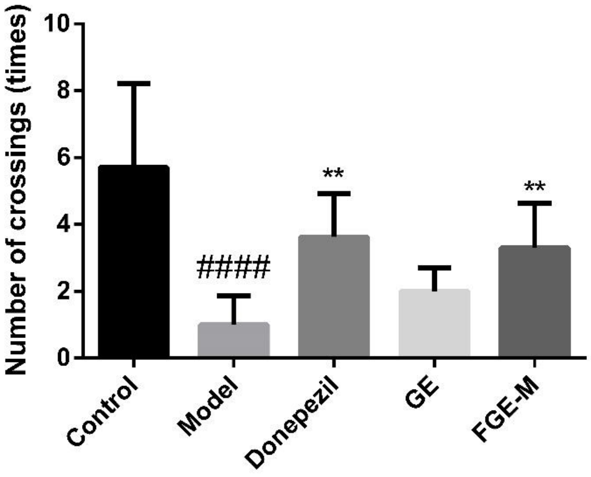

Figure S1: Effects of GE powder and FGE on number of crossings of AD-like mice. GE (240 mg/kg b. w.), FGE-M (240 mg/kg b. w.). Data are expressed as means ± SD (n = 10) of six independent experiments. ####p＜0.0001, compared with control group, **p＜0.05, compared with model group

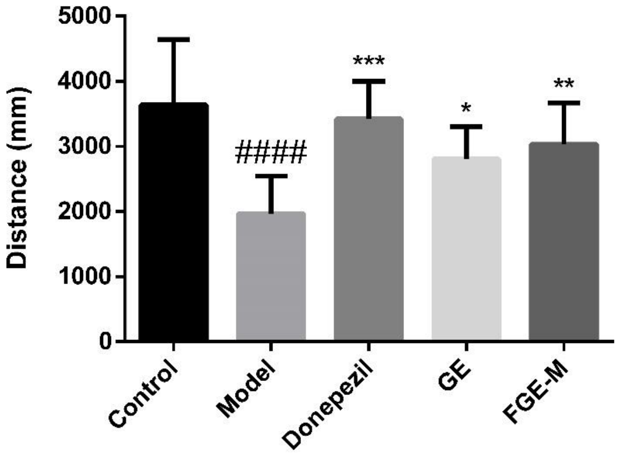

Figure S2: Effects of GE powder and FGE on total movement distance of AD-like mice. GE (240 mg/kg b. w.), FGE-M (240 mg/kg b. w.). Data are expressed as means ± SD (n = 10) of six independent experiments. ####p＜0.0001, compared with control group, ***p＜0.001, **p＜0.05, compared with model group.

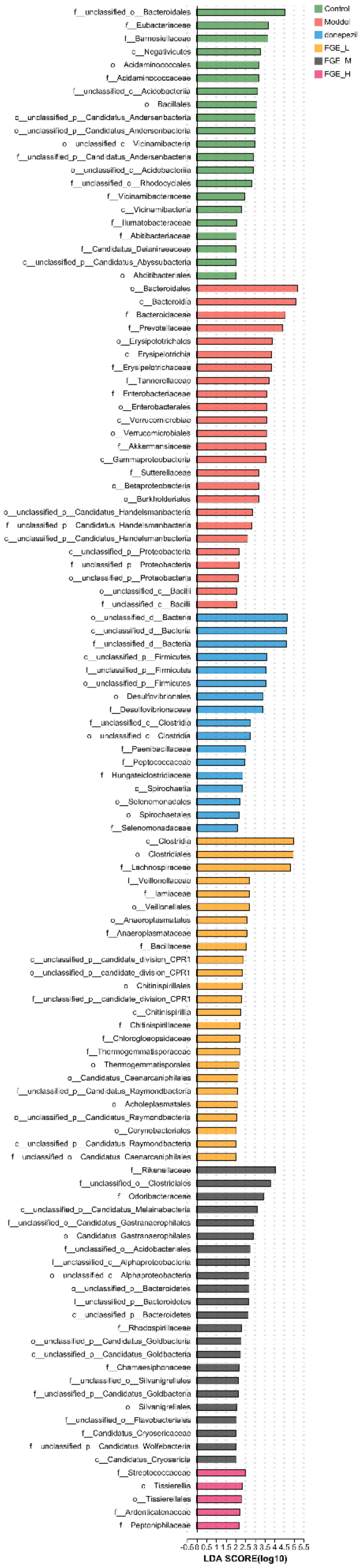

Figure S3: LDA Effect Size analysis of community differences between groups.

Table S1: Chemical composition identification of FGE using HR-LC/MS in positive mode.

Compounds	Rt (min)	$[M+H]^+$ m/z	M^+ (Calc.)
**Maltose**	0.92	343.1242	342.1162
**Kojic acid**	0.98	142.2225	142.0266
***p*-hydroxybenzyl alcohol**	1.65	124.0394	124.0524
**Benzyl 3-chlorophenyl ether**	1.71	219.0504	218.0498
**Apocynin**	1.72	166.0862	166.0630
***p*-ethoxybenzyl alcohol**	1.88	152.0837	152.0837
***p*-hydroxybenzoic acid**	1.93	138.0317	138.0317
**1-furan-2-yl-2-(4-hydroxyphenyl)-ethanone**	2.37	203.1389	202.0630
***p*-hydroxybenzaldehyde**	2.44	122.0368	122.0368
**Ethyl succinate**	2.54	175.0579	174.0892
**Ethyl *p*-hydroxybenzoate**	2.57	166.0630	166.0630
**4-Chloro-alpha-(3,4-xylyl)-o-cresol**	2.81	247.0811	246.0811
**4,4'-sulfinylbis(methylene) diphenol**	2.82	263.0771	262.0664
**4, 4'-Dihydroxyd iphenyl methane**	2.90	201.0786	200.0837
**Cirsiumaldehyde**	2.92	234.0872	234.0528
**1,3-Diphenylacetone**	3.14	211.1075	210.1045
**2,4,4'-Trihydroxybenzophenone**	3.40	231.1701	230.0579
**4-Isopropylphenol**	3.78	137.0233	136.0888
**1,3-di(furan-2-yl)prop-2-en-1-one**	4.30	199.0760	198.1256
**Bungein A**	4.37	274.1205	274.1205
**4,4'-Dihydroxybenzyl sulfone**	4.78	315.1502	314.0824
**Dibenzyl sulfoxide**	4.92	231.1702	230.0765
**Bis (4-hydroxybenzyl) sulfide**	5.80	247.1175	246.0715
**4-(1-Methylbutyl) phenol**	5.81	165.0547	164.1201
**3,4-Bis-(p-hydroxyphenyl)-2,4-hexadien**	7.24	267.1545	266.1307
**4-(((4-ethoxybenzyl)oxy)methyl)-phenol**	7.80	259.1145	258.1256
**Hexestrol**	7.96	270.1923	270.1620
**4-(4'-Hydroxybenzyloxy) benzyl methyl ether**	9.22	245.1895	244.1099
**N-Valylphenylalanine**	9.86	265.2072	264.1474
**(2R)-2-(3-chloro-4-methylsulfonylphenyl)-3-cyclopentyl-N-pyrazin-2-ylpropanamide**	9.94	407.2386	407.1070
**Benzestrol**	10.89	299.0977	298.1933
**L-Leu-L-Glu**	11.82	261.2071	260.1372
**L-Ile-L-Pro-L-Ile**	12.47	342.2386	341.2315
**4-hydroxy-3-(4-hydroxybenzyl) benzyl methyl ether**	12.89	245.0100	244.1099
**Bisphenol F**	13.24	201.1120	200.0837
**4,4'-Dihydroxybiphenyl**	13.40	189.0968	188.0837
**3-((4-hydroxybenzyl)oxy) propane-1,2-diol**	15.54	198.0908	198.0892

**L-Leu-L-Pro**	16.07	229.0865	228.1474
**Ethyl 1-benzylpyridin-1-ium-3-carboxylate**	17.44	243.1022	242.1181
**4-hydroxy 3–(4-hydroxybenzyl) benzyl methyl ether**	17.94	259.2015	258.1256
**1,3-di (furan-2-yl) prop-2-en-1-one**	18.07	189.0500	188.0473
**Gastrodin**	18.48	287.1749	286.1053
**9(10)-EpOME**	19.34	297.2264	296.2351
**4,4'-Dithiodianiline**	19.47	248.2741	248.0442
**4-butoxyphenylmethanol**	19.61	181.0708	180.1150
**Strobilactone A**	20.33	367.2097	266.1518
**Bisphenol A**	21.08	228.1647	228.1150
**9-Oxo-10*E*,12*Z*-octadecadienoic acid**	21.24	295.2266	294.2195
**Parishin D**	21.25	404.2700	404.1107
**2,4-bis(4-hydroxybenzyl) phenol**	22.07	304.2014	303.1256
**4-(ethoxymethyl)-glucopyranosyl-phenol**	24.09	315.2539	314.1366
***p*-hydroxyphenethyl alcohol**	43.26	168.0746	167.0946
**Acetyl sulfide**	43.69	118.0089	118.0089
